# Vitamin D Supplementation and Mental Health in Inflammatory Bowel Diseases and Irritable Bowel Syndrome Patients: A Systematic Review

**DOI:** 10.3390/nu13103662

**Published:** 2021-10-19

**Authors:** Dominika Głąbska, Aleksandra Kołota, Katarzyna Lachowicz, Dominika Skolmowska, Małgorzata Stachoń, Dominika Guzek

**Affiliations:** 1 Department of Dietetics, Institute of Human Nutrition Sciences, Warsaw University of Life Sciences (WULS-SGGW), 159C Nowoursynowska Street, 02-776 Warsaw, Poland; aleksandra_kolota@sggw.edu.pl (A.K.); katarzyna_lachowicz@sggw.edu.pl (K.L.); dominika_skolmowska@sggw.edu.pl (D.S.); malgorzata_stachon@sggw.edu.pl (M.S.); 2 Department of Food Market and Consumer Research, Institute of Human Nutrition Sciences, Warsaw University of Life Sciences (WULS-SGGW), 159C Nowoursynowska Street, 02-776 Warsaw, Poland; dominika_guzek@sggw.edu.pl

**Keywords:** inflammatory bowel diseases (IBD), irritable bowel syndrome (IBS), vitamin D, supplementation, supplement, mental health, disease-specific quality of life, quality of life, anxiety, depression

## Abstract

Inflammatory bowel diseases (IBDs) and irritable bowel syndrome (IBS) are associated with decreased quality of life and mental health problems. Among various approaches to supportive therapy that aims to improve mental health in affected individuals, vitamin D supplementation is considered to be an effective method which may also be beneficial in alleviating the symptoms during the course of IBDs and IBS. The aim of the present study was to conduct a systematic review of the literature presenting the data regarding the influence of vitamin D supplementation on mental health in adults with inflammatory and functional bowel diseases, including IBDs and IBS. This study was conducted in accordance with the Preferred Reporting Items for Systematic Reviews and Meta-Analyses (PRISMA) guidelines and registered in the International Prospective Register of Systematic Reviews (PROSPERO) database (Registration number CRD42020155779). A systematic search of the PubMed and Web of Science databases was performed, and the intervention studies published until September 2021 were included. The human studies eligible to be included in the review should have described any intervention involving vitamin D as a supplement in a group of adult patients suffering from IBDs and/or IBS and should have assessed any component of mental health, but studies presenting the effects of combined supplementation of multiple nutrients were excluded. After eliminating the duplicates, a total of 8514 records were screened and assessed independently by two researchers. Further evaluation was carried out on the basis of title, abstract, and full text. Finally, 10 studies (four for IBDs and six for IBS) were selected for the current systematic review, and their quality was assessed using the Newcastle–Ottawa Scale (NOS). The studies analyzed the influence of various doses of vitamin D on bowel diseases, compared the results of vitamin D supplementation with placebo, or administered specific doses of vitamin D to obtain the required level in the blood. Supplementation was performed for at least 6 weeks. The analyzed mental health outcomes mainly included disease-specific quality of life/quality of life, anxiety, and depression. The majority of studies (including high-quality ones) confirmed the positive effect of vitamin D supplementation on the mental health of IBD and IBS patients, which was proven by all research works evaluating anxiety and depression and by the majority of research works evaluating quality of life. Although the studies followed different dosage regimens and supplementation protocols, the positive influence of vitamin D on mental health was found to be consistent. The number of studies on patients suffering from ulcerative colitis and the availability of trials randomized against the placebo group was low in the current review, which is considered to be a limitation of the present study and could also reflect the final outcome of the analysis. The conducted systematic review established the positive effect of vitamin D supplementation on the mental health of IBD and IBS patients, but this result requires further investigation, particularly in relation to other mental health outcomes.

## 1. Introduction

Inflammatory bowel diseases (IBDs), including ulcerative colitis (UC) and Crohn’s disease (CD), are idiopathic intestinal disorders that are characterized by repetitive episodes of inflammation of the gastrointestinal tract, usually associated with bloody diarrhea, tenesmus, and abdominal pain, while the location of the disease and the thickness of the affected bowel wall differ in the diseased individuals [1]. They are characterized by alternating periods of remissions and relapses [2] and predicting the exact course of the disease is difficult [3]. IBDs exert a significant influence on the quality of life of the affected patients, which was demonstrated by a recent systematic review and meta-analyses by Knowles et al. [4], who claims that the quality of life of IBD patients depends on the stage and type of disease, which is found to be lower during relapses than during remissions and lower in CD than in UC. Similarly, the incidence and prevalence of psychiatric disorders are higher in IBD patients than in the general population [5]. Mental health problems in IBD patients, as it was proven for depression and anxiety, may be associated with the symptoms or the course of the disease or may be related to the diagnostic procedures [6], and are found to be quite common [7].

The diagnostic problems in IBDs are mainly due to the lack of appropriate diagnostic tools to differentiate between UC and CD [8], as well as to distinguish IBDs from other gastrointestinal diseases, including irritable bowel syndrome (IBS) [9]. IBS is the condition that is diagnosed in the absence of any other causative disease, and is based on recurrent abdominal pain or discomfort with altered bowel habits [10]. Similar to IBD, this disorder is also associated with decreased quality of life, as confirmed by the recent systematic review by El-Serag et al. [11]. Moreover, the systematic review and meta-analysis by Lee et al. [12] showed that depression and anxiety levels in IBS patients are higher than the levels observed in healthy controls.

As described above, the IBDs and IBS may influence the mental health of patients, and their mental health may further influence the somatic course of the disease, which is observed in both IBD [13] and IBS patients [14]. Therefore, attempts to improve the mental health of patients with inflammatory and functional bowel diseases would be of great benefit to them.

Among the various methods of supportive therapy that are known to positively impact the mental health of patients, nutrition is considered to be one of the promising and potential options to reduce the symptoms of depression, as demonstrated in the systematic review and meta-analysis by Firth et al. [15] and systematic review by Ljungberg et al. [16], as well as to reduce the symptoms of depression and anxiety in the systematic review by Saha et al. [17]. In addition, diet is indicated as an important element of pathogenesis and therapy for both IBDs [18] and IBS [19], so dietary intervention may be a probable therapeutic option to effectively reduce mental health problems and improve the quality of life within a routine nutrition management program.

Of the various dietary factors known to play a key role in alleviating mental health problems, vitamin D is one of the most effective components. The meta-analyses by Vellekkatt & Menon [20], Shaffer et al. [21], and Spedding [22] established that vitamin D supplementation reduces depression. Furthermore, a meta-analysis by Cheng et al. [23] showed that this nutrient helps in coping with negative emotions, and a systematic review by Hoffmann et al. [24] indicated that it improves the quality of life. Moreover, this supportive therapy may be promising for IBD and IBS patients in particular, as vitamin D supplementation was confirmed to reduce the relapse rate of IBD [25] and improve IBS symptom severity scores [26].

Taking into consideration the above-described state of knowledge, the present study aimed to conduct a systematic review of the literature analyzing the influence of vitamin D supplementation on the mental health status of adults with inflammatory and functional bowel diseases, including IBDs and IBS.

## 2. Materials and Methods

### 2.1. The Registration and Design

The systematic review was conducted in accordance with the Preferred Reporting Items for Systematic Reviews and Meta-Analyses (PRISMA) guidelines [27], based on the recommendations for systematic literature search, screening, inclusion, and reporting. This study was registered in the International Prospective Register of Systematic Reviews (PROSPERO) database (Registration number CRD42020155779). The methodology followed in this systematic review was similar to the protocol adopted for evaluating the influence of vitamin D on the mental health of children [28]. A systematic search of PubMed and Web of Science databases was conducted in two stages. The first stage included studies common for the present systematic review and the preceding one [28] (studies published until October 2019) and the second stage included supplementary articles (studies published from October 2019 to September 2021). The search was restricted to the studies published in the English language in peer-reviewed journals.

### 2.2. The Assessment of Eligibility and Inclusion to a Systematic Review

The studies eligible to be included in a systematic review of influence of vitamin D supplementation on mental health in adults with inflammatory and functional bowel diseases were ought to describe any intervention including any dose of vitamin D supplemented and the assessment of any component of mental health.

The following inclusion criteria were scheduled:

(1) adult population studied;
(2) patients with confirmed IBDs and/or IBS studied;
(3) applied vitamin D dose defined within the study;
(4) any component of mental health assessed within the study (assessed using either subjective or objective measurements).

The following exclusion criteria were scheduled:

(1) animal model studies;
(2) studies conducted in populations with concurrent intellectual disabilities;
(3) studies conducted in populations with concurrent eating disorders;
(4) studies conducted in populations with concurrent neurological disorders, including Alzheimer’s disease, epilepsy, etc.;
(5) conducted assessment of supplementation of multiple nutrients combined;
(6) studies conducted in populations of mothers/children, analyzing the association between maternal vitamin D supplementation and the mental health of their offspring.

No other exclusion criteria, associated with the stage or course of disease, studied population, or country were scheduled.

### 2.3. The Electonic Search Strategy and Procedure

The detailed description of electronic search strategy is presented in Supplementary Table S1, separately for PubMed and Web of Science databases.

After conducting an electronic search and identifying potentially eligible studies, the duplicates were removed and the studies were verified based on the previously planned inclusion and exclusion criteria. They were verified in three phases—on the basis of title, abstract, and full text. If the full text was not available, the corresponding author of the study was asked for the full text of their study. The assessment in each phase was conducted independently by two researchers, while the results of the searching were verified by comparison of their lists and if any disagreement appeared, it was consulted by third researcher.

**Figure 1:**
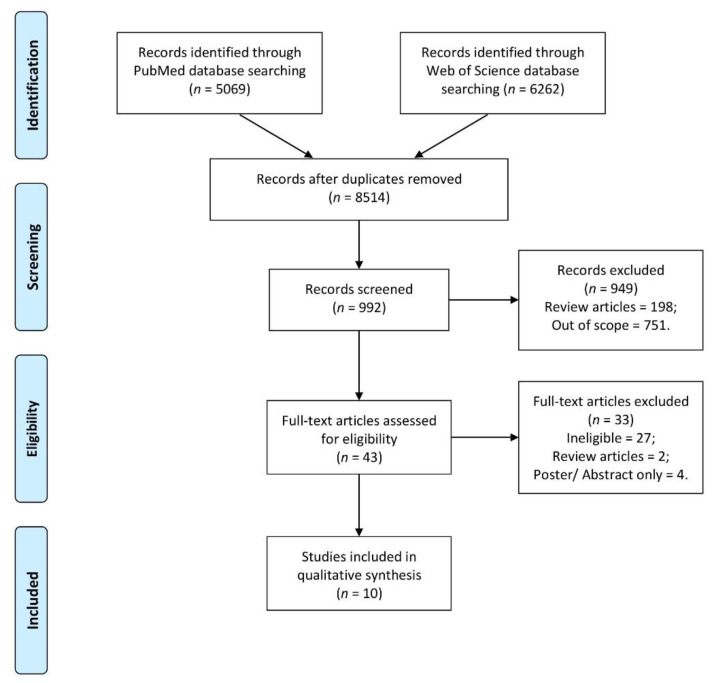
The detailed procedure of including studies.

The detailed procedure of including studies is presented in Figure 1.

### 2.4. The Data Extraction Procedure

The data extraction was conducted based on the standard procedure, while researchers extracted the following data:

(1) the basic characteristics of the studies and of the studied populations (authors and year; design of the study; country/location; studied group; time);
(2) the detailed characteristics of the patients studied (participants number; female participants number; age; inclusion criteria; exclusion criteria);
(3) the detailed characteristics of the applied vitamin D supplementation intervention and mental health outcome (vitamin D measure; applied vitamin D supplementation; mental health outcome; psychological measure);
(4) the observations and conclusions (observations; conclusions).

If any information was not available, the corresponding author of the study was asked about the details of their study (data marked in the systematic review as provided on request). The data were extracted independently by 2 researchers, while the extracted data were verified by comparison of their results and if any disagreement appeared, it was consulted by third researcher.

The included studies were assessed in terms of the quality of the study, expressed as the risk of bias, as recommended by Cochrane [29]. The Newcastle-Ottawa Scale (NOS) was used as a preferred tool to assess the quality of non-randomized studies [30]. The following criteria were taken into consideration: selection (score: from 0 to 4), comparability (score: from 0 to 2), and exposure/outcome (score: from 0 to 3), while the studies were attributed to the following categories: very high risk of bias (total score: from 0 to 3), high risk of bias (total score: from 4 to 6), and low risk of bias (total score: from 7 to 9) [31].

## 3. Results

### 3.1. The Studies Conducted in Inflammatory Bowel Disease Patients

The basic characteristics of the studies and of the studied populations of IBD patients for the studies included to the systematic review [32,33,34,35] are presented in Table 1. The studies of IBD patients included in the systematic review were conducted mainly in CD patients [32,34], but one study was conducted in UC patients [35] and one in a mixed population comprising both CD and UC patients [33]. The studies were performed in the United States of America [32,33], Canada [34], and Iran [35], and the study samples were recruited by the universities/university medical centers [32,33,34] or hospital/private gastroenterology clinics [35].

The detailed characteristics of the studied IBD patients for the studies included to the systematic review are presented in Table 2. This systematic review included studies of IBD patients that were conducted mainly in small samples of patients (less than 50 individuals) [32,34,35], but also involved one large study comprising almost 1000 individuals [33]. The patients were included in the studies based on their confirmed IBD diagnosis [32,33,34,35], defined disease activity/remission [32,34,35], defined serum 25(OH)D level [32,35], or nonconsumption of vitamin D supplements prior to the study [34,35].

The detailed characteristics of the applied vitamin D supplementation intervention and mental health outcomes for the studies of the IBD patients included to the systematic review are presented in Table 3. The studies of IBD patients included in the systematic review mainly compared the efficacy of various doses of vitamin D [34,35] or applied such doses to obtain the required vitamin D blood level [32,33]. The intervention period ranged from a minimum of 12 weeks [33,34,35] and extended up to 24 weeks [32]. The assessed mental health outcomes mainly included disease-specific quality of life, which was analyzed using the Inflammatory Bowel Disease Questionnaire (IBDQ) [32,35] or Short Inflammatory Bowel Disease Questionnaire (SIBDQ) [33], while anxiety and depression were analyzed using the Hospital Anxiety and Depression Scale (HADS) [34]. The detailed description of observations and conclusions for the IBD studies included to the systematic review is presented in Table S2.

### 3.2. The Studies Conducted in Irritable Bowel Syndrom Patients

The basic characteristics of the studies and of the studied populations of IBS patients for the papers included in the systematic review [36,37,38,39,40,41] are presented in Table 4. The studies included in the present review were conducted in the general population comprising IBS patients [36,37,38,39,41], but one study was conducted in a specific population of diarrhea-predominant IBS patients [40]. The studies were carried out in Iran [37,38,39,40] and the United Kingdom [36,41], and the study subjects were recruited by the universities/university medical centers [36,37,41] or hospitals/gastroenterology clinics [38,39,40].

The detailed characteristics of the studied IBS patients for the papers included in the systematic review are presented in Table 5. The studies of IBS patients were conducted in medium-size samples consisting of 50–150 patients [36,37,38,39,40,41]. The patients were included in the studies based on their confirmed IBS diagnosis [36,37,38,39,40,41], defined disease severity score [40], defined serum 25(OH)D level [39,40], or nonconsumption of vitamin D supplements prior to the study [36,37,38,39,40,41].

The detailed characteristics of the applied vitamin D supplementation intervention and mental health outcomes for the studies of the IBS patients included in the systematic review are presented in Table 6. The intervention studies of IBS patients selected for this review evaluated the efficacy of different doses of vitamin D and compared the results with placebos [36,37,38,39,40,41], and the intervention period lasted for a minimum of 6 weeks [36,38,39,40,41] or extended to even 6 months [37]. The mental health outcomes that were analyzed in all these studies mainly included disease-specific quality of life, which was evaluated using the Irritable Bowel Syndrome Quality of Life questionnaire (IBS-QoL) [37,38,39,40,41], the general quality of life, assessed by asking simple question [36], and anxiety and depression, evaluated using the Hospital Anxiety and Depression Scale (HADS) [40]. The detailed description of observations and conclusions for the IBS studies included to the systematic review is presented in Table S3.

### 3.3. The Summary of Studies Conducted in Inflammatory Bowel Disease and Irritable Bowel Syndrome Patients

The summary of the observed association between vitamin D supplementation and mental health outcomes for the studies of the IBD and IBS patients included in the systematic review, accompanied by the NOS total score, is presented in Table 7. While assessing the quality of the studies, the majority (six studies) were associated with a low risk of bias [35,37,38,39,40,41], while five of the high-quality studies supported the positive influence of vitamin D supplementation on mental health outcomes for IBD [35] and IBS [37,38,39,40,41]. Moreover, among the studies reviewed, only one study did not support the notion that vitamin D supplementation exerts a positive impact on mental health outcomes [41], and two studies did not provide any conclusion, as they did not present the results regarding the influence of vitamin D supplementation on any component of mental health [33,36]. While many studies were performed to determine the effect of vitamin D on various mental health outcomes, it must be emphasized that two studies evaluated the influence of vitamin D supplementation on anxiety and depression and established its positive impact [34,40]. In addition, the majority of studies also confirmed the positive influence of this nutrient on quality of life [32,35,37,38,39,40].

## 4. Discussion

The conducted systematic review confirmed the proposed hypothesis that vitamin D supplementation positively influences the mental health of bowel disease patients. Only one study did not support this positive influence [41], and all other studies, which published their findings, supported this assumption [32,34,35,37,38,39,40]. The observations are in agreement with the previous conclusions by Williams et al. [26], who conducted a review of various studies on IBS patients and proved the influence of vitamin D supplementation on the symptoms of disease, including the quality of life. Despite the fact that the authors of the reviewed studies specified that larger and adequately powered interventions are needed to establish the therapeutic application of vitamin D in IBS patients, they suggested that vitamin D interventions may be beneficial in enhancing the quality of life of the affected individuals [26]. This result is confirmed by the present review conducted specifically to determine the influence of vitamin D on the mental health of patients suffering from IBS and IBDs, which showed that the intake of this vitamin provides significant benefits for mental health outcomes. However, it should be emphasized that only the quality of life/disease-specific quality of life [32,33,35,36,37,38,39,40], anxiety, and depression were assessed in these works so far [34,40]. Moreover, the number of studies representing the IBD population was also low, and only one study included a defined population of UC patients [35]. Similarly, only one study clearly defined IBS patients as diarrhea-predominant [40]. Hence, in order to obtain more conclusive results, it is necessary to include more studies that are performed in a specific group of population and should be based not only on the incidence of IBD or IBS but also on the type of disease and its clinical course.

As the majority of studies included in this systematic review assessed the disease-specific quality of life/quality of life, it can be confirmed that this mental health outcome is significantly associated with the disease course and symptoms, as revealed in both IBD [4] and IBS patients [42]. Thus, the beneficial effects of vitamin D may be manifested by two mechanisms—either a direct impact on mental health or an indirect influence, wherein vitamin D shows a positive influence on the symptoms of disease [25,26] and thereby improves the quality of life [4,42]. As indicated in a systematic review by Hoffmann et al. [24], the influence of vitamin D supplementation on the health-related quality of life in various diseases is still not explored well. Nevertheless, the current evidence indicates that vitamin D supplementation may have a small-to-moderate effect on the quality of life when taken for a short period of time [24]. This observation was further confirmed by the present systematic review that included studies conducted in specific populations of IBD and IBS patients.

Moreover, it is of particular importance that two studies assessing the influence of vitamin D on anxiety and depression, in IBD [34] and IBS patients [40] confirmed the positive influence of the applied supplementation. As anxiety and depression are found to be common mental health problems associated with various diseases, including the COVID-19 global pandemic, as revealed by a systematic review by Xiong et al. [43], the approaches to reduce them is of great importance. In addition, the World Health Organization (WHO) declared depression as one of the major health conditions that need to be given top priority, due to its major impact not only on the patient’s health but also on their personal and social life [44]. Henceforth, for bowel disease patients who are already suffering from the disease, any attempt to reduce anxiety and depression symptoms would be of considerable advantage.

However, except for the mental health outcomes, for which the influence of vitamin D supplementation has been studied so far, there are some reports revealing that bowel diseases are also associated with other mental health outcomes, which include self-esteem [45], loneliness [46], feeling worried [47], stress [48], and suicidal behavior in IBD patients [49], as well as well-being [50], self-esteem [51], feeling worried [52], stress [53], hopelessness [54], and suicidal behavior in IBS patients [55]. There is an urgent need to conduct studies to determine whether vitamin D supplementation can improve the other mental health conditions associated with IBD or IBS.

The results indicate that vitamin D supplementation may be beneficial to alleviate the symptoms during the course of bowel disease [25,26] and may exert a positive influence on mental health [21,22,23,24], but there is a serious concern with regard to the deficiency of this nutrient worldwide [56]. Therefore, supplementation is an effective approach for the appropriate management of vitamin D deficiency in the global population [57].

In spite of the fact that the conducted systematic review highlighted some novel observations for the betterment of IBD and IBS patients, some limitations of the study need to be acknowledged. The most important issue is the inclusion of a small number of studies, especially the small number of studies randomized against placebo, and that no studies randomized against a placebo representing the IBD population were included. Moreover, it should be emphasized that there is a risk of overlap in the results presented within the included studies, since two studies [38,39] were conducted by the same team and followed a similar experimental protocol, so there is a possibility for the same subjects to have been included in both of them.

## 5. Conclusions

The conducted systematic review confirmed the positive influence of vitamin D supplementation on the mental health of bowel disease patients, observed for both IBD and IBS. The majority of the studies supported the beneficial effect of vitamin D supplementation on the studied mental health outcomes, such as disease-specific quality of life/quality of life, anxiety, and depression. Though the studies adopted different vitamin D dosage regimens for varied periods of time, the general observation of positive effect on mental health is consistent. However, the limited number of studies selected for the review, especially for UC, must be considered as a limitation of the present analysis. The effect should be further studied in a larger sample of patients and on other mental health outcomes.

## Figures and Tables

**Table 1:** The basic characteristics of the studies and of the studied populations of inflammatory bowel disease (IBD) patients for the studies included to the systematic review.

Ref.	Authors, Year	Design of the Study	Country/Location	Studied Group	Time
[32]	Yang et al., 2013	Open labelled, prospective clinical trial	United States of America (USA)/Pennsylvania	Patients with confirmed Crohn’s disease from Pennsylvania State University	Not specified
[33]	Kabbani et al., 2016	Longitudinal study	United States of America (USA)/Pittsburgh	Patients with Inflammatory Bowel Diseases from the University of Pittsburgh Medical Center	From 1 January 2009 to 31 December 2013
[34]	Narula et al., 2017	Randomized, double-blind placebo-controlled trial	Canada/Ontario	Patients with confirmed Crohn’s disease from McMaster University Medical Centre in Hamilton	From January 2014 to March 2015
[35]	Karimi et al., 2019	Randomized, double-blind clinical trial	Iran/Tehran	Patients with mild to moderate Ulcerative Colitis with vitamin D deficiency referring to Shahid Fayyaz-Bakhsh Hospital, and a private gastroenterology clinic	Not specified

**Table 2:** The detailed characteristics of the studied inflammatory bowel disease (IBD) patients for the studies included to the systematic review.

Ref.	Participants (Female Participants) Number	Age (Mean ± SD/Range)	Inclusion/Exclusion Criteria
[32]	18 (11)	38.0 ± 17.0 years	Inclusion: aged 18–70; confirmed Crohn’s disease; CDAI scores 150–400; serum levels of 25(OH)D < 40 ng/mL Exclusion: ulcerative colitis; any other inflammatory bowel conditions; ostomy; receiving corticosteroid therapy
[33]	965 (505)	44.0 ± 10.1 years	Inclusion: confirmed inflammatory bowel disease; being patient at the University of Pittsburgh Medical Center from 1 January 2009 to 31 December 2013 Exclusion: -
[34]	34 (20)—at baseline	According to group: 35.0 ± 3.0. years (low-dose of vitamin D supplementation) 33.0 ± 3.0 years (high-dose of vitamin D supplementation)	Inclusion: aged 18–70, diagnosis of Crohn’s disease; clinical remission for at least 28 days with a HBI of ≤ 4; maintenance therapies for Crohn’s disease at a stable dose for at least 3 months; used vitamin D supplements at the time of enrolment discontinued for a period of at least 6 weeks Exclusion: systemic steroid therapy in the preceding 4 weeks; pregnancy or considering pregnancy during the study period; short-gut syndrome; any condition which could predispose to vitamin D toxicity, including renal insufficiency, sarcoidosis, hyperparathyroidism, or malignancy; therapy with thiazide diuretics, barbiturates, digitalis, or supplemental products containing vitamin D
[35]	46 (22)	According to group: 39.7 ± 15.6 years (low-dose of vitamin D supplementation) 34.0 ± 12.5 years (high-dose of vitamin D supplementation)	Inclusion: aged ≥ 18 years; histopathologic diagnosis of mild to moderate ulcerative colitis (diagnosis of the severity based on physician’s judgment); vitamin D deficiency (<75 nmol/L); no change in the type and dosage of their medicine over the past month Exclusion: other diseases; intestinal disorders; known autoimmune diseases; cancer; inflammatory and infectious diseases; using vitamin D supplements, mineral-multivitamins, omega-3, polyphenolic and antioxidant medications, anticoagulants, non-steroid anti-inflammatory drugs, antihistamines and calcium channel antagonists during the past month; pregnancy; breastfeeding; in women—using contraceptives; changes in the type and dosage of the drugs during the study

CDAI—Crohn’s Disease Activity Index; HBI—Harvey-Bradshaw Index.

**Table 3:** The detailed characteristics of the applied vitamin D supplementation intervention and mental health outcomes for the studies of the inflammatory bowel disease (IBD) patients included to the systematic review.

Ref.	Vitamin D Measure	Intervention—Vitamin D Supplementation	Mental Health Outcome	Psychological Measure
[32]	25(OH)D vitamin D level in blood Three 24-h dietary recalls	25–125 µg/day for 24 weeks while 25(OH)D vitamin D level in blood was controlled each 2 weeks and in case of result of <100 nm/L, the doses of vitamin D were gradually increased	Disease-specific quality of life	IBDQ
[33]	25(OH)D vitamin D level in blood	25(OH)D vitamin D level in blood was controlled each 2 weeks and in case of result of <75 nm/L, the supplementation of vitamin D was applied at dose of 1250 µg/week or 2 weeks, for at least 12 weeks	Disease-specific quality of life	SIBDQ
[34]	25(OH)D vitamin D level in blood	25 µg vs. 250 µg/day for 12 months	Anxiety and depression	HADS
[35]	25(OH) vitamin D levels in blood Three 24-h dietary recalls	25 µg vs. 50 µg/week for 12 weeks	Disease-specific quality of life	IBDQ-9

25-hydroxyvitamin D (25(OH)D); IBDQ—The Inflammatory Bowel Disease Questionnaire; SIBDQ—Short Inflammatory Bowel Disease Questionnaire; HADS—Hospital Anxiety and Depression Scale.

**Table 4:** The basic characteristics of the studies and of the studied populations of irritable bowel syndrome (IBS) patients for the studies included to the systematic review.

Ref.	Authors, Year	Design of the Study	Country/Location	Studied Group	Time
[36]	Tazzyman et al., 2015	Randomized, double-blind, placebo-controlled, stratified study	United Kingdom (UK)/Sheffield	Patients with IBS recruited at the University of Sheffield	From January 2014 to April 2014
[37]	Abbasnezhad et al., 2016	Randomized, double-blind placebo-controlled clinical trial	Iran/Ahvaz	Patients with IBS from the outpatient clinic at the Jundishapur University of Medical Sciences	From February to March 2015
[38]	Jalili et al., 2016	Factorial blinded randomized clinical trial	Iran/Tehran	Women with IBS from the Endoscopy Clinic, Shariati Hospital	From 2013
[39]	Jalili et al., 2019	Randomized, double-blind, placebo-controlled clinical trial	Iran/Tehran *	Women with IBS recruited from two gastroenterology clinics	From October 2013 to January 2016
[40]	Sikaroudi et al., 2020	Randomized, double-blind, placebo-controlled trial study	Iran/Tehran	Patients with IBS-D recruited from Rasoul-e-Akram Hospital	February 2017 to May 2018
[41]	Williams et al., 2021	Randomized, double-blind, placebo-controlled study	United Kingdom	Patients with IBS recruited through the University of Sheffield, via the IBS Network	December 2017 to March 2019

* data provided on request; IBS—Irritable Bowel Syndrome; IBS-D—diarrhea-predominant Irritable Bowel Syndrome.

**Table 5:** The detailed characteristics of the studied irritable bowel syndrome (IBS) patients for the studies included to the systematic review.

Ref.	Participants (Female Participants) Number	Age (Mean ± SD/Range)	Inclusion/Exclusion Criteria
[36]	51 (47)	According to group: 34 ± 12 years (vitamin D supplementation) 36 ± 15 years (placebo)	Inclusion: confirmed IBS (the ROME III criteria) Exclusion: any antibiotic use in the past 4 weeks; recent changes in IBS medication; pregnancy; current use of vitamins or probiotic supplements; history of gastrointestinal surgery; diabetes; current use of antidepressants or antipsychotics
[37]	85 (57)	37.9 years (range 18–73)	Inclusion: aged 18–70; confirmed IBS (the ROME III criteria) Exclusion: any evidence of abdominal surgery or radiation; celiac disease, or other primary GI illnesses; GI infection obscuring IBS symptoms; total parenteral nutrition therapy in the last 6 months; pregnancy; lactation; alcohol consumption; concurrent chronic diseases such as diabetes, renal failure, and kidney stones; diagnosed and/or treated malignancy in the past 5 years; serum calcium levels > 10.6 mg/dL; intake of vitamin D, omega-3, vitamin E, and calcium supplements; being on a special diet or medication regimen during the last 6 months
[38]	100 (100)	According to group: 41.32 ± 12.62 years (vitamin D and placebo of soy isoflavones supplementation) 39.76 ± 12.99 years (placebo of vitamin D and of soy isoflavones supplementation)	Inclusion: women; aged 18–75; confirmed IBS (the ROME III criteria); BMI of 18–25 kg/m^2^ Exclusion: intestinal organic diseases; intestinal infection; history of colorectal disorders; major intestinal surgery; current use of antibiotics; anti-diarrhea and anti-constipation drugs; non-steroidal anti-inflammatory drugs; metocloperamide, cisaperide, difenoxilate, opium and immune suppressors; use of any type of soy products and/or vitamin D; use of synthetic sweeteners 2 days before and during the study; pregnancy; lactation; history of breast cancer (in case of patient, her mother and sisters); diet therapy; hormone therapy; substantial changes in dietary intakes during the study; using vitamin D supplements or soya supplements (not planned within intervention) during the study
[39]	116 (116)	According to group: 42.24 ± 12.26 years (for vitamin D supplementation) 40.06 ± 13.37 years (for placebo)	Inclusion: women; aged 18–75; confirmed IBS (the ROME III criteria); BMI of 18–25 kg/m^2^ Exclusion: intestinal organic diseases; intestinal infection; history of colorectal disorders; major intestinal surgery; current use of antibiotics; anti-diarrhea and anti-constipation drugs; non-steroidal anti-inflammatory drugs; metocloperamide, cisaperide, difenoxilate, opium and immune suppressors; use of any type of soy products and/or vitamin D; use of synthetic sweeteners 2 days before and during the study; pregnancy; lactation; history of breast cancer (in case of patient, her mother and sisters); diet therapy; hormone therapy; substantial changes in dietary intakes during the study; using vitamin D supplements or soya supplements (not planned within intervention) during the study; serum vitamin D level of > 75 nmol/L
[40]	74 (39)	35.51 ± 10.43 years	Inclusion: aged 18–65 years; confirmed IBS (the ROME IV criteria and World Gastroenterology Organization questionnaire for healthcare professional of IBS patients criteria); IBS-SSS score of 175–300 Exclusion: pregnancy; lactation; GI disorders such as inflammatory bowel disease, celiac disease, GI infection; history of colon cancer, intestinal surgery or radiotherapy, and cholecystectomy; taking vitamin D supplement in the last 6 months; use of other supplements; nonsteroidal anti-inflammatory drugs, glucocorticoid and antidepressants drug containing serotonin resorptive antagonists, selective serotonin reuptake inhibitors, tricyclic antidepressants used; alcohol, or caffeine intake or smoking 12 h before the laboratory test; serum vitamin D > 75 nmol/L; any abnormal response or side effect to supplementation; blood in the stool; fast weight lost; using < 80% of supplements
[41]	135 (106)	30.01 ± 10.46 years	Inclusion: aged ≥ 18 years; confirmed IBS (the ROME III or ROME IV criteria at diagnosis); a TSS ≥ 150 Exclusion: regular use of nutritional supplements; pregnancy; lactation; BMI < 18 kg/m^2^; BMI > 30 kg/m^2^; any history of other gastrointestinal disorders (e.g., inflammatory bowel diseases, diverticulitis, cancer); diabetes; recent or planned vacation

IBS—Irritable Bowel Syndrome; GI—gastrointestinal; BMI—Body Mass Index; (IBS-SSS)—Irritable Bowel Syndrome—Severity Score System; TSS—Total Symptom Severity Score.

**Table 6:** The detailed characteristics of the applied vitamin D supplementation intervention and mental health outcomes for the studies of the irritable bowel syndrome (IBS) patients included to the systematic review.

Ref.	Vitamin D Measure	Intervention—Vitamin D Supplementation	Mental Health Outcome	Psychological Measure
[36]	25(OH)D vitamin D level in blood FFQ	75 µg/day vs. placebo for 12 weeks	Quality of life	Simple question (“How much has IBS affected your life?”)
[37]	25(OH)D vitamin D level in blood	1250 µg/2 weeks vs. placebo for 6 months	Disease-specific quality of life	IBS-QoL
[38]	Three 24-h dietary recalls	1250 µg/2 weeks vs. placebo for 6 weeks	Disease-specific quality of life	IBS-QoL
[39]	25(OH)D vitamin D level in blood 3-days dietary recall	1250 µg/week vs. placebo for 6 weeks	Disease-specific quality of life	IBS-QoL
[40]	25(OH) vitamin D level in blood 3-day dietary records	1250 µg/week vs. placebo for 9 weeks	(1) Disease-specific quality of life (2) Anxiety and depression	(1) IBS-QoL (2) HADS
[41]	25(OH) vitamin D level in blood	75 µg/day vs. placebo for 12 weeks	Disease-specific quality of life	IBS-QoL

25-hydroxyvitamin D (25(OH)D); FFQ—Food Frequency Questionnaire; IBS-QoL—Irritable Bowel Disease-Quality of Life questionnaire; HADS—Hospital Anxiety and Depression Scale.

**Table 7:** The summary of observed association between vitamin D supplementation and mental health outcomes for the studies of the inflammatory bowel disease (IBD) and irritable bowel syndrome (IBS) patients included to the systematic review, accompanied by the Newcastle-Ottawa Scale (NOS) total score.

Studied Group	Ref.	Observed Association between Vitamin D Supplementation and Mental Health Outcomes		Quality of the Study ^b^
		Outcome	The Association—Supporting/Inconclusive/Not Supporting ^a^	
Inflammatory bowel diseases	[32]	Disease-specific quality of life	Supporting	5
	[33]	Disease-specific quality of life	Inconclusive	3
	[34]	Anxiety and depression	Supporting	6
	[35]	Disease-specific quality of life	Supporting	8
Irritable bowel syndrome	[36]	Quality of life	Inconclusive	5
	[37]	Disease-specific quality of life	Supporting	7
	[38]	Disease-specific quality of life	Supporting	7
	[39]	Disease-specific quality of life	Supporting	9
	[40]	Disease-specific quality of life, anxiety and depression	Supporting	9
	[41]	Disease-specific quality of life	Not supporting	7

^a^ Supporting—positive influence of applied vitamin D supplementation concluded for any component of mental health; inconclusive—no results of the influence of applied vitamin D supplementation conducted for any component of mental health presented; not supporting—no positive influence of applied vitamin D supplementation concluded for any component of mental health; ^b^ the Newcastle-Ottawa Scale (NOS) total score to be interpreted within the following categories: very high risk of bias (0–3 NOS points), high risk of bias (4–6 NOS points), and low risk of bias (7–9 NOS points) [31].

## Supplementary Materials

The following are available online at https://www.mdpi.com/article/10.3390/nu13103662/s1. Table S1: The detailed description of electronic search strategy for PubMed and Web of Science databases. Table S2: The detailed description of observations and conclusions for the inflammatory bowel disease (IBD) studies included to the systematic review. Table S3: The detailed description of observations and conclusions for the irritable bowel syndrome (IBS) studies included to the systematic review.
