# Supplementary material for: Vitamin D Supplementation and Mental Health in Inflammatory Bowel Diseases and Irritable Bowel Syndrome Patients: A Systematic Review
Source: Nutrients. 2021 Oct 19;13(10):3662. doi: 10.3390/nu13103662 (PMC8540769; doi:10.3390/nu13103662)
Supplement: Supplementary file 1 [file nutrients-13-03662-s001.zip › nutrients-1398200-supplementary.pdf]

nutrients

MDPI

Supplementary Materials

The Vitamin D Supplementation and Mental Health in Bowel Diseases Patients: A Systematic Review

Dominika Głąbska [1,*], Aleksandra Kołota [1], Katarzyna Lachowicz [1], Dominika Skolmowska [1], Małgorzata Stachoń [1], and Dominika Guzek [2]

Supplementary Table S1. The detailed description of electronic search strategy for PubMed and Web of Science databases.

Database	Electronic Search Strategy for PubMed and Web of Science databases
PubMed	(((mental health[Title/Abstract] OR mental disorders[Title/Abstract] OR mental disorder[Title/Abstract] OR psychological distress[Title/Abstract] OR mood disorders[Title/Abstract] OR depression[Title/Abstract] OR suicidal[Title/Abstract] OR suicide[Title/Abstract] OR anxiety[Title/Abstract] OR well-being[Title/Abstract] OR wellbeing[Title/Abstract] OR quality of life[Title/Abstract] OR self esteem[Title/Abstract] OR self-esteem[Title/Abstract] OR self efficacy[Title/Abstract] OR self-efficacy[Title/Abstract] OR resilience[Title/Abstract] OR empowerment[Title/Abstract] OR social participation[Title/Abstract] OR mental capital[Title/Abstract] OR life skills[Title/Abstract] OR emotional[Title/Abstract] OR psychology[Title/Abstract] OR psychosocial[Title/Abstract] OR psychiatry[Title/Abstract])) AND (vitamin D[Title/Abstract] OR vitamin D2[Title/Abstract] OR vitaminD3[Title/Abstract] OR D2[Title/Abstract] OR D3[Title/Abstract] OR ergocalciferol[Title/Abstract] OR cholecalciferol[Title/Abstract] OR 25-hydroxyvitamin D[Title/Abstract] OR 3-epi-25hydroxyvitaminD[Title/Abstract] OR calcitriol[Title/Abstract] OR dihydroxycholecalciferol[Title/Abstract])) NOT (animal NOT (animal AND human)[MeSH Terms])
Web of Science	(TS=('vitamin D' OR 'vitamin D2' OR 'vitamin D3' OR 'D2' OR 'D3' OR 'ergocalciferol' OR 'cholecalciferol' OR '25-hydroxyvitamin D' OR '3-epi-25 hydroxyvitamin D' OR 'calcitriol' OR 'dihydroxycholecalciferol') AND TS=('mental health' OR 'mental disorders' OR 'mental disorder' OR 'psychological distress' OR 'mood disorder' OR 'depression' OR 'suicidal' OR 'suicide' OR 'anxiety' OR 'well-being' OR 'wellbeing' OR 'quality of life' OR 'self esteem' OR 'self-esteem' OR 'self efficacy' OR 'self-efficacy' OR 'resilience' OR 'empowerment' OR 'social participation' OR 'mental capital' OR 'life skills' OR 'emotional' OR 'psychology' OR 'psychosocial' OR 'psychiatry') NOT TS=('animal' NOT ('animal' AND 'human')))

Supplementary Table S2. The detailed description of observations and conclusions for the inflammatory bowel disease (IBD) studies included to the systematic review.

Ref.	Observations	Conclusions
[34]	Quality-of-life scores improved following vitamin D supplementation ($p = 0.0004$).	Vitamin D supplementation of patients with mild-to-moderate Crohn's disease is effective at improving the quality of life.
[35]	Subjects with low vitamin D levels had worse pain, disease activity scores, and quality of life ($p < 0.05$), but it was not studied for supplementation.	Subjects who received vitamin D supplements had a significant reduction in their health-care utilization, but it was not studied for quality of life.
[36]	Improvement in anxiety and depression scores and a good safety profile were observed in both groups treated with vitamin D3.	Patients receiving either dose of vitamin D3 demonstrated improvement in mood, and the dose of 250 μg daily showed promise for normalizing mood symptoms in those with clinical anxiety and depression.
[37]	IBDQ-9 mean score significantly increased in high dose group compared to the low dose group ($p = 0.001$).	Our results indicate that 50 μg daily dose of vitamin D can increase quality of life, while it reduces disease activity in UC patients with vitamin D deficiency.

IBDQ – The Inflammatory Bowel Disease Questionnaire; UC – ulcerative colitis.

Supplementary Table S3. The detailed description of observations and conclusions for the irritable bowel syndrome (IBS) studies included to the systematic review.

Ref.	Observations	Conclusions
[38]	Applied treatment did not influence quality of life at any of the time point.	The impact of IBS on quality of life may be reduced by higher vitamin D level, but it was not proven for supplementation.
[39]	The IBS-QoL scores in the vitamin D group significantly improved compared to the placebo group postintervention (14.26 ± 3.0 vs 11.0 ± 2.34, $p < 0.001$).	Vitamin D seems to be an effective and safe option to improve QoL in patients with IBS.
[40]	The vitamin D supplementation significantly improved the IBS-QOL scores (The adjusted mean of IBS-QOL score was 35.48 for patients who were received vitamin D, while the adjusted mean for patients who were not received it was 44.04, *p*-value = 0.015)	Our findings have revealed that co-administration of soy isoflavones with vitamin D could not improve the IBS-QOL scores, although each treatment improved significantly the quality of life.
[41]	IBS-QOL at week 6 was improved significantly more in Vitamin D group in comparison to placebo group.	This study indicates that Vitamin D therapy can improve the severity of symptoms and QOL in female patients with IBS; however, the long-term effects remained to be elucidated.
[42]	The QoL, and HADS-depression improved significantly in the vitamin D group as compared to the placebo group (*p*-values: 0.049 and 0.023, respectively). There were no significant differences in abdominal HADS-anxiety between the two groups at the end of the study.	Based on our results, we recommend serum vitamin D be evaluated in the process of treatment of these patients to ameliorate symptoms and quality life of IBS-D patients with vitamin D deficiency and/or insufficiency.
[43]	There was no difference between trial arms (vitamin D supplementation vs. placebo) in the change in quality of life (− 7.7 ± 25.36 vs – 11.31 ± 25.02, $p = 0.427$).	This study found no benefit of vitamin D supplementation on QoL measures using standardized assessments.

IBS – Irritable Bowel Syndrome; IBS-D – diarrhea-predominant Irritable Bowel Syndrome; IBS-QoL – Irritable Bowel Disease-Quality of Life questionnaire; HADS – Hospital Anxiety and Depression Scale; QoL – Quality of Life questionnaire.
